# Immunization of Mice with Anthrax Protective Antigen Limits Cardiotoxicity but Not Hepatotoxicity Following Lethal Toxin Challenge

**DOI:** 10.3390/toxins7072371

**Published:** 2015-06-25

**Authors:** T. Scott Devera, Dawn K. Prusator, Sunil K. Joshi, Jimmy D. Ballard, Mark L. Lang

**Affiliations:** 1 Department of Microbiology and Immunology, University of Oklahoma Health Sciences Center, Oklahoma City, OK 73104, USA; E-Mails: tscottdev@gmail.com (T.S.D.); dawn-prusator@ouhsc.edu (D.K.P.); skjoshi@odu.edu (S.K.J.); jimmy-ballard@ouhsc.edu (J.D.B.); 2 Oklahoma Center for Neuroscience, University of Oklahoma Health Sciences Center, Oklahoma City, OK 73104, USA; 3 Frank Reidy Research Center for Bioelectrics, Old Dominion University, Norfolk, VA 23508, USA

**Keywords:** *Bacillus anthracis*, lethal toxin, protective antigen, neutralizing antibody, troponin

## Abstract

Protective immunity against anthrax is inferred from measurement of vaccine antigen-specific neutralizing antibody titers in serum samples. In animal models, *in vivo* challenges with toxin and/or spores can also be performed. However, neither of these approaches considers toxin-induced damage to specific organ systems. It is therefore important to determine to what extent anthrax vaccines and existing or candidate adjuvants can provide organ-specific protection against intoxication. We therefore compared the ability of Alum, CpG DNA and the CD1d ligand α-galactosylceramide (αGC) to enhance protective antigen-specific antibody titers, to protect mice against challenge with lethal toxin, and to block cardiotoxicity and hepatotoxicity. By measurement of serum cardiac Troponin I (cTnI), and hepatic alanine aminotransferase (ALT), and aspartate aminotransferase (AST), it was apparent that neither vaccine modality prevented hepatic intoxication, despite high Ab titers and ultimate survival of the subject. In contrast, cardiotoxicity was greatly diminished by prior immunization. This shows that a vaccine that confers survival following toxin exposure may still have an associated morbidity. We propose that organ-specific intoxication should be monitored routinely during research into new vaccine modalities.

## 1. Introduction

*Bacillus anthracis* secretes an 83 kDa protein, known as protective Ag (PA), that forms heptameric pores on the surface of target cells expressing anthrax toxin receptors (capillary morphogenesis protein-2, CMG-2 and tumor endothelial marker-8,TEM-8) [1,2]. PA heptamers interact with lethal factor (LF) and edema factor (EF) to form lethal toxin (LT) and edema toxin (ET), respectively, which together are known as anthrax toxin [2]. The PA heptamer facilitates entry of EF and LF into the target cell. Within the target cell LF functions as a zinc-dependent metalloprotease and cleaves mitogen activated protein kinase kinases [3]. Following cell entry, EF, which has calmodulin-dependent adenylate cyclase activity, generates high concentrations of cAMP [4]. LT and ET have potent immune-subversive effects during the early stages of infection, inhibiting the functions of dendritic cells, B cells, T cells and NKT cells [5,6,7,8]. In later stages of infection, anthrax toxin is lethal in animal models and causes a broad range of defects in target cells, including altered cell cycle, cell growth and survival [9]. A recent study using organ-specific conditional knockout of the TEM-8 and CMG-2 receptors, demonstrated that LF largely asserts its lethality on the heart, despite intoxication of multiple organ systems [10]. In contrast, EF largely causes lethality via the liver and gastrointestinal system [10].

Collectively the activities of anthrax toxin cripple the host and allow *B. anthracis* to grow to high numbers in the bloodstream [2,5,6,7,8,10]. Hence, immune neutralization of PA counters the damaging effects of anthrax toxin, providing protection to the host during early stages of disease. PA-specific Ab neutralizes anthrax toxin *in vitro* and protects immunized animals *in vivo* following a lethal challenge [11,12,13,14,15,16]. There is a strong correlation between PA-specific Ab titers and toxin neutralization by sera from patients who have survived *B. anthracis* infection [17]. Consequently, there is considerable interest in development of vaccines, which incorporate PA as the immunogen [17,18]. The current vaccine anthrax vaccine-adsorbed (AVA) administered to US military personnel consists of PA and induces PA-specific Ab titers sufficient to neutralize anthrax toxin [17,18]. However, what is not well-appreciated is the extent to which PA-specific Ab protects the cardiac and the hepatic systems from intoxication. In light of the new studies showing that LF mediates lethality via the heart [10], it is important to evaluate the effects of immunization on intoxication.

Herein, we show that immunization of mice with PA induced Ab titers that were protective against cardiotoxicity and lethality following *in vivo* challenge with LT. However, anti-PA Abs did not protect against hepatotoxicity. This pattern was also observed when different types of adjuvant were included in the PA vaccine. This work suggests that anthrax vaccination might protect the host from lethality, but not from all sources of organ damage and associated morbidity.

## 2. Results

### 2.1. Anthrax Toxin Leads to Elevation of Serum Hepatic AST and ALT and Cardiac Troponin I in Naïve Mice

**Figure 1:**
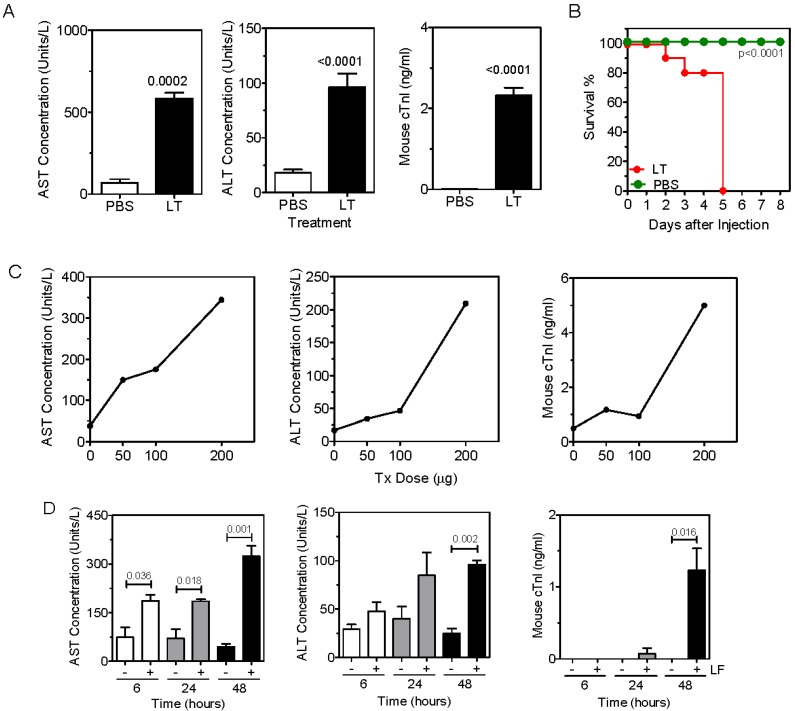

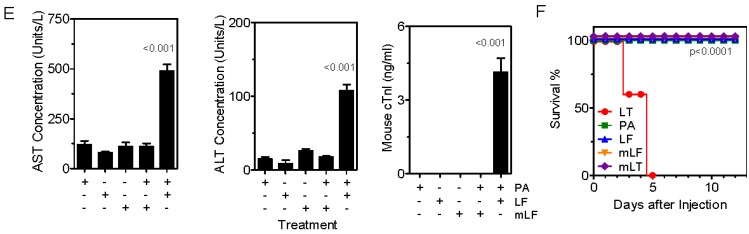
Anthrax toxin leads to elevation of serum hepatic AST and ALT and cardiac Troponin I in naïve mice. (**A**) C57Bl/6 mice were treated with LT in PBS or PBS alone and blood samples collected after 48 h. AST, ALT and Troponin I concentrations were then measured. Data show the mean ± SEM enzyme concentration (*n =* 10 per group); (**B**) Mice were treated with either LT in PBS or PBS alone. Mice were observed for times indicated and survival monitored (*n =* 10 per group); (**C**) Mice were treated with the doses of LT indicated and rested for 48 h before collecting blood samples and measuring AST, ALT and cTnI. Each data point represents an individual mouse; (**D**) Mice were treated with LT and blood samples were collected at times shown. Data shows the mean ± SEM serum AST, ALT and cTnI enzyme concentrations (*n =* 3 per group); (**E**) Mice were treated with PA, LF, mutant lethal factor (mLF), mLT or LT and blood samples were collected at 48 h. AST, ALT and cTnI were then measured. (**F**) Survival was monitored (*n =* 5 per group). Statistical significance in (**A**) and (**D**) were determined by Mann Whitney U-test, and in (**E**) by one-way ANOVA and Bonferroni’s multiple comparisons post-test. Statistical significance in (**B**) and **(F**) was determined by performing Kaplan-Meier analysis in conjunction with a log rank test. The p values indicate significant differences between survival of the PBS- and the LT-treated groups in (**B**), and between all groups and the LT-treated group in (**F**).

Following an i.v. challenge of naïve C57Bl/6 mice with LT, a clear elevation in serum concentrations of hepatic AST and ALT, as well as cardiac Troponin I (cTN1) was observed (Figure 1A). Mice treated with LT ultimately succumbed to intoxication and had 0% survival 5 days after toxin administration (Figure 1B). Elevation of serum concentrations of AST, ALT and cTn1 was dose-dependent with respect to LT (Figure 1C). Intoxication was most evident 48 hours after treatment with LT but could be observed after 24 hours (Figure 1D). Intoxication and lethality of LT required both the PA protein and enzymatically-active LF. Administration of either protein alone, or PA combined with an inactive LT mutant failed to cause elevation of AST, ALT, cTn1 (Figure 1E) or result in death (Figure 1F). These results demonstrate that LT administration led to hepatotoxicity, as well as cardiotoxicity.

### 2.2. Immunization with PA Induces Ab and Inhibits Cardiotoxicity but not Hepatotoxicity

**Figure 2:**
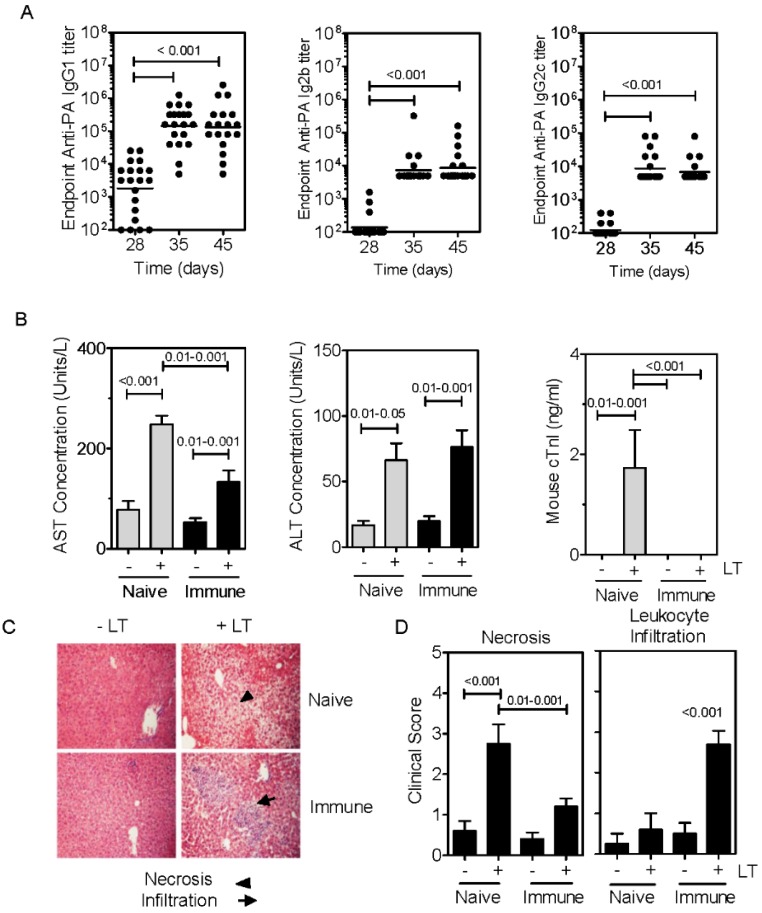
Immunization with PA induces PA-specific Ab and inhibits cardio- but not hepato-toxicity. (**A**) C57Bl/6 mice were immunized with 8 µg of PA in PBS on day 0 and boosted with 5 µg of PA on day 28. Mice were bled on days indicated. Data show PA-specific IgG1 (left), IgG2b (center) and IgG2c (right) Ab titers. Each data point represents an individual mouse; (**B**) Naïve and PA-immunized mice were treated with LT and blood samples collected after 48 h. AST, ALT and Troponin I were then measured. Data show the mean ±SEM serum enzyme concentrations (*n =* 5 for naïve group, *n =* 10 for immunized group). Two outliers (non-responders with value = 0) were removed from the naïve challenged group in the cTn1 assay; (**C**) Liver sections were prepared from parallel groups of mice and stained with H&E; (**D**) Slides were scored for leukocyte infiltration and coagulative necrosis (*n =* 5 per group). Statistical significance in (**A**,**B**) and (**D**) was determined by one-way ANOVA and Bonferroni’s multiple comparisons post-test.

Following immunization of mice with PA, anti-PA IgG1, IgG2b and IgG2c titers were readily detected in the serum (Figure 2A). This is consistent with numerous published reports [12,16,19,20,21]. When naïve and PA-immunized mice were challenged i.v. with LT, significant AST elevations were observed in naïve and immunized mice, albeit of a lesser magnitude in the immunized group (Figure 2B). There was no statistical difference in the elevations of serum ALT in naïve and immunized groups. Additionally, elevations in cTn1 were effectively blocked in all mice in the PA-immunized group (Figure 2B). Closer examination of hepatotoxicity revealed a modest but significant decline in coagulative necrosis in the PA-immunized mice (Figure 2C,D). However, an increase in leukocyte infiltration was evident in LT-challenged PA-immunized mice as compared to other groups (Figure 2D). These data show that immunization of mice with PA resulted in an Ab response that blocked the cardiotoxicity but had only minor effects on the hepatotoxicity associated with LT.

### 2.3. Inclusion of Adjuvants in the PA Vaccine Does not Affect LT-Induced Hepatotoxicity 

**Figure 3:**
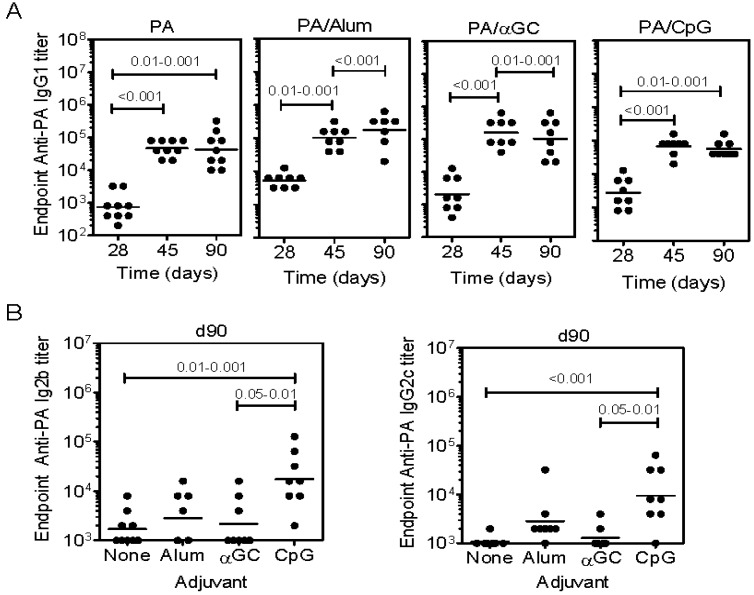

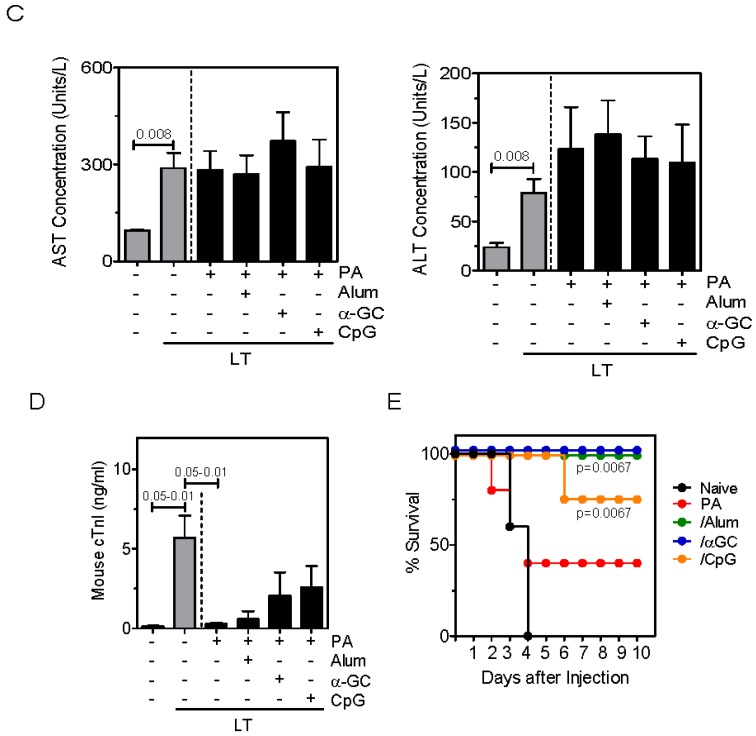
Effects of adjuvant inclusion on PA-specific Ab titers and LT-induced toxicity. Mice were immunized and bled according to schedule in Figure 2 using the formulations indicated (**A**) Shows PA-specific IgG1 titers; (**B**) Shows PA-specific IgG2b and IgG2c titers. 9–10 mice per group were immunized. Naïve and immunized mice were bled and then treated with high dose LT split over two doses (200 µg PA plus LF followed by 100 µg PA plus LF 24 h later). Statistical significance in (**A**) and (**B**) was determined by one-way ANOVA and Bonferroni’s multiple comparisons post-test; (**C**) ALT and AST were measured as described (*n =* 3–5 mice per group). One-way ANOVA did not reveal significant differences between groups in (**C**). The p value shown in (**C**) results from Mann-Whitney U test comparison of the naïve/unchallenged and naïve/LT-challenged groups; (**D**) Troponin I concentrations were then measured as described (*n =* 3–5 mice per group). One way ANOVA revealed significant differences between the means and *p* value ranges indicate results of post-test with Bonferroni’s correction applied; (**E**) Shows survival of naïve and immunized mice (*n =* 4–5 mice per group). Statistical significance was determined by performing Kaplan-Meier analysis in conjunction with a log rank test. The *p* values indicate significant differences between survival of the indicated groups and the naïve group.

Anti-PA titers were compared when PA was administered alone, adsorbed to Alum adjuvant, or mixed with αGC or CpG DNA (Figure 3A,B). Although PA alone was immunogenic, IgG1 titers were modestly increased by inclusion of adjuvants (Figure 3A). Ab responses were dominated by IgG1, except in the case of CpG DNA which induces a Th1-polarized response, consistent with the higher IgG2b and IgG2c titers observed (Figure 3B).

Immunized mice and additional naïve controls were challenged with LT before collecting blood samples, measuring AST, ALT (Figure 3C) and cTn1 (Figure 3D). Neither of the adjuvants used affected LT-induced elevations in serum AST or ALT. As observed in the previous experiment, immunization with PA alone reduced elevation of serum cTn1. The inclusion of adjuvants led to a variable cTn1 response in those experimental groups, which was not significantly different from controls. It should be noted that some mice succumbed to toxin before collecting serum samples.

Survival was monitored and as expected, all naïve mice succumbed to LT within 4 days (Figure 3D). There was a 40% survival rate in the PA-immunized mice, an 80% survival rate in the CpG-immunized mice, and a 100% survival rate in the Alum- or α-GC-immunized mice (Figure 3E).

These data show that the vaccine adjuvants tested did not enhance Ab responses in a manner that increased protection against LT-induced cardiotoxicity or hepatotoxicity as compared to immunization with PA alone. However, inclusion of adjuvants did improve overall survival, thus delineating morbidity and lethality.

## 3. Discussion

It was reported that LF and EF result in lethality in mice via the cardiac and hepatic systems respectively [10]. This presents the important question of how cellular intoxication is affected following vaccination with PA. It is well recognized that immunization with PA leads to anthrax toxin-neutralizing PA-specific IgG. It is also appreciated that PA-specific IgG can bind the protein at different sites, affecting interaction with the CMG-2 and TEM-8 host receptors, can affect PA polymerization to form the heptameric pore, and can affect the interaction with LF and/or EF [22,23,24]. Some IgG could also be deleterious, facilitating transport of LF and/or EF into target cells, thus worsening intoxication. It still remains unclear which IgG sub-classes confer protection. Casadevall and colleagues demonstrated that a monoclonal IgG2a Ab directed against PA conferred better *in vivo* protection than the same clone engineered as an IgG1 mAb [25]. Through the use of IL-4^−/−^ and IFN^−/−^ mice lacking the capacity to produce IgG1 or IgG2c (IgG2a equivalent in B6 mice), we observed that polyclonal IgG1 was a major protective IgG sub-class [21]. IgG Fc receptors (FcγR) contribute to protection [25], and different FcγR may do so differentially although a rank order of importance of each receptor *in vivo* has not been established.

Despite the advances in understanding how Ab interacts with toxin and the effector arm of the immune system, the effects of PA-specific IgG on organ-specific intoxication remain less-well explored. In one study, LT-challenged canines showed improved survival and cardiac function when receiving hemodynamic support in combination with anti-PA mAb therapy [26]. Herein we report the surprising observation that immunization with PA, while protective against LT-induced lethality, limited cardiotoxicity but not hepatotoxicity. This was evidenced as immunized mice having undetectable serum cTN1 concentrations but elevated serum ALT and AST following LT challenge. In contrast, non-immunized controls had elevated cTn1, ALT and AST. The results therefore suggest that circulating PA-specific Ab has a selective ability to protect different organs from intoxication. 

Although IgG titers and survival were improved by inclusion of adjuvants in the vaccine, they did not lead to inhibition of the ALT and AST elevation following challenge with LT. This included Alum adjuvant which is used in the AVA vaccine as well as experimental adjuvants including CD1d-binding ligand αGC and TLR9-activating CpG DNA. Since immunization with PA alone efficiently blocked cardiotoxicity, it was not surprising that inclusion of adjuvants did not further enhance this effect. However, as mentioned, adjuvants did boost survival following challenge with a high dose of LT. Although the IgG1 titers were modestly (2–4 fold) enhanced by Alum or α-GC, there was a profound difference in survival. This suggests that a threshold of high affinity Ab that binds neutralizing epitopes must be attained to permit survival. Our study involved the administration of relatively high amounts of LT, but was consistent with other challenge studies where typically 3–4 fold more protein is administered to the relatively LT-resistant B6 mice than the more susceptible BALB/c or A/J mice [7,20,27,28,29]. The amount of LT administered in previous challenge studies appears to exceed that which has previously been detected in the bloodstream of infected guinea pigs and mice [30,31]. However, Tang, and colleagues demonstrated that most (>95%) of LT administered to mice by the intravenous route cannot be detected in the sera as little as two hours after treatment and is only 2–3 fold higher than that detected after challenge with 10^7^ live Ames 35 strain spores [31]. It therefore appears that inactivation and clearance of LT delivered as an intravenous bolus (especially in immunized animals) is likely to remove or neutralize much of the LT that was originally administered. This notion is supported by Tang and colleagues, who demonstrated detection of higher amounts of an un-cleavable form of PA after using it for challenge (PA-U7) [31]. Therefore, the measured concentrations of PA in the serum following natural infection cannot be assumed to be discordant with the amount of protein used for LT challenge experiments.

Our data raise the question of why would circulating PA-specific IgG protect the heart from intoxication but not protect the liver. LT and ET cause lethality via expression of the high affinity PA receptor CMG-2 on cardiac myocytes and hepatocytes respectively [10]. Differential expression of CMG-2 could therefore be a factor in the remaining hepatotoxicity. It is also possible that because circulating IgG immune complexes are cleared by the liver in an Fc receptor-dependent manner [32,33], a good IgG titer that protects the heart also directs some IgG/LT complexes to the liver that is able to still bind CMG-2 and intoxicate hepatocytes.

Since LT lethality does not appear to be mediated by the liver [10], one could assume that the remaining hepatotoxicity does not present a problem or limitation of the PA-based vaccine. However, residual hepatotoxicity mediated by ET following natural infection could lead to morbidity even in PA-vaccinated individuals, or perhaps predispose to later hepatic disease. A system-wide assessment of organ-specific protection following toxin challenge would be a worthwhile endeavor and perhaps prompt consideration of complementary vaccine strategies that offer a more comprehensive protection of the host.

## 4. Materials and Methods

### 4.1. Reagents

BL21 competent cells were purchased from Invitrogen (Carlsbad, CA). The pET15b plasmids encoding PA and LF respectively were a gift from Dr. J. Collier (Harvard Medical School) and have been described previously [1]. HRP-conjugated anti-IgG1, -IgG2b and -IgG2c were purchased from Southern Biotechnology (Birmingham, AL, USA). The αGC ligand was purchased from Axorra Inc. (San Diego, CA, USA). Imject Alum was purchased from Pierce Biotechnology (Rockford, IL, USA). CpG DNA (ODN1826) was purchased from Invivogen (San Diego, CA, USA). Kits for measurement of Aspartyl aminotransferase (AST) and Alanine aminotransferase (ALT) were purchased from Bio Scientific Corp. (Austin, TX, USA). The cardiac Troponin 1 (cTn1) kit was purchased from Life Diagnostics Inc. (West Chester, PA, USA).

### 4.2. Toxin Expression and Purification

Histidine-tagged PA and LF were expressed separately in competent BL21 *Escherichia coli* (Invitrogen, Carlsbad, CA, USA) transformed with the pET15b-rPA, pET15b-rLF and pET15brLF^H690C^ plasmids respectively. PA and LF were then purified from bacterial lysates using standard methods also described previously [34]. In brief, PA and LF purification was achieved using a 5 mL HisTrap affinity column (GE Life Sciences, Piscataway, NJ, USA). LPS contamination was removed by from purified PA and LF using an EndoTrap Red LPS-binding affinity column (Lonza, Walkersville, MD, USA).

### 4.3. Mice 

Female C57Bl/6 mice were purchased from the National Cancer Institute (Bethesda, MD, USA). Experiments were performed on mice aged 6–10 weeks.

### 4.4. Ethics Statement

All animal procedures reported herein were approved by the University of Oklahoma Health Sciences Center Institutional Animal Care and Use Committee (IACUC) and work performed under protocol (11-120-HI) in accordance with guidelines established by the NIH Office of Laboratory Animal Welfare.

### 4.5. Retro-Orbital Eye Bleed and Serum Collection

Mice were anesthetized using a vaporized 4% isofluorane/96% oxygen mixture and 100 μL blood collected by retro-orbital bleed with heparinized micro-capillary tubes (Fisher Scientific, Hampton, NH, USA). Samples were transferred immediately to polypropylene micro-centrifuge tubes. Blood samples were incubated for 30 min at room temperature then allowed to clot overnight at 4 °C, before centrifugation at 13,000 *g* for 15 min at 4 °C. Sera were withdrawn with a pipette and stored in aliquots at −20 °C.

### 4.6. Immunizations and Experimental Schedule 

A single subcutaneous (s.c.) immunization was administered over both flanks on day 0 immediately following collection of pre-bleed sera. Unless indicated otherwise, immunizations consisted of 8 μg PA in 200 μL sterile-endotoxin-free PBS or PA mixed with 4 μg of αGC, 5 µg of CpG DNA, or adsorbed to 100 µL of Imject Alum. Mice were then bled at day 28 post-immunization and sera obtained. On day 28, mice were bled and then boosted s.c. with 5 μg of PA in PBS and bled on day 45 and day 90. At the end of the experimental period, mice were either challenged with toxin or euthanized in order to obtain liver tissue.

### 4.7. In Vivo Toxin Challenge

The LF and PA subunits were mixed at a 1:1 molar ratio in PBS. An amount of lethal toxin equivalent to 200 μg PA and in a 100 μL volume was then administered by the i.v. para-orbital route to mice. Procedure associated-deaths precluded the use of anesthesia for *in vivo* challenge assays. This protocol was approved by the IACUC and a qualified veterinarian (S.K.J.) administered the toxin. Where indicated, after 24 h a second dose equivalent to 100 μg PA was administered. When PA, LF, or mutant LF were administered separately, 200 µg of protein was used. The mice were then monitored daily for the duration of the experiment and time to death or moribund-based euthanasia recorded.

### 4.8. ELISA for Serum Ig 

Immulon 4 ELISA–plates (Dynex Technologies Inc. Chantilly, VA, USA), were coated with PA at 10 μg/mL in binding buffer (0.1 M Na_2_HPO_4_, pH 9.0), overnight at 4°C before washing plates and blocking for 2 h at room temperature with 1.0 % w/v BSA in PBS/0.05% v/v Tween 20. Sera were diluted 100, 1000, or 10,000 fold in PBS/0.05% v/v Tween as appropriate and then subjected to 2-fold serial dilutions. Dilutes sera was then added to PA-coated, pre-blocked plates. Plates were incubated overnight at 4 °C with diluted sera, before washing 4 times in PBS/0.05% v/v Tween 20. Plates were incubated for 1 h at room temperature with HRP-conjugated anti-mouse IgG1, IgG2b or IgG2c at a final concentration of 0.2 μg/mL. Plates were washed and developed for 5 min at room temperature using 90 μL of ABTS substrate per well (KPL, Gaithersburg, MD, USA). Reactions were stopped by addition of 110 μL of a 10% w/v SDS solution. Plates were analyzed using a Dynex MRX Revelation plate reader. Endpoint titers were determined as O.D. <0.01 at 405 nm (equivalent to O.D. of 1/200 dilution of pre-bleed sera). Individual Ab titers were plotted as geometric means using GraphPad Prism software. A non-parametric Mann-Whitney U test was used to assess experiments with two experimental groups. Multiple experimental groups were assessed by one-way ANOVA with Dunn’s post-test.

### 4.9. AST, ALT, and cTn1 Measurement 

Serum concentrations of AST, ALT and cTn1 were measured using commercial assay kits according to manufacturers’ instructions.

### 4.10. Histological Analysis of Liver

Livers were collected from terminally-moribund animals after euthanasia and from groups treated with LT or PBS. Livers were preserved in 10% neutral-buffered formalin for 24 h then dehydrated and embedded in paraffin. Tissues were then sectioned and stained with hematoxylin and eosin and subjected to microscopic analysis. Scoring for LT-associated pathology was performed in a blinded fashion.
